# Change in Purpose in Life Before and After Onset of Cognitive Impairment

**DOI:** 10.1001/jamanetworkopen.2023.33489

**Published:** 2023-09-13

**Authors:** Angelina R. Sutin, Martina Luchetti, Yannick Stephan, Antonio Terracciano

**Affiliations:** 1 Florida State University College of Medicine, Tallahassee; 2 Euromov, University of Montpellier, Montpellier, France

## Abstract

**Importance:**

Purpose in life is a critical aspect of psychological well-being that is associated with better cognitive outcomes across the continuum of dementia. To our knowledge, the natural history of purpose with onset of cognitive impairment has yet to be evaluated.

**Objective:**

To evaluate changes in purpose in life prior to and after onset of cognitive impairment.

**Design, Setting, and Participants:**

This cohort study used assessments of purpose in life and cognitive status from March 2006 to May 2021 in the Health and Retirement Study (HRS) and from May 2011 to November 2021 in the National Health and Aging Trends Study (NHATS).

**Exposure:**

Cognitive impairment at each wave based on established thresholds in HRS and NHATS.

**Main Outcomes and Measures:**

The main outcome was purpose in life, measured with the Ryff Measures of Psychological Well-Being in HRS and a validated item in NHATS.

**Results:**

In HRS, 22 668 participants provided 50 985 assessments of purpose across all waves. In NHATS, 10 786 participants provided 53 880 assessments of purpose across all waves. In HRS, 58.3% of participants were female, with mean (SD) age of 64.76 (10.41) years at baseline; in NHATS, 57.4% were female, with mean (SD) age of 76.82 (7.71) years at baseline. Across waves, 6794 HRS participants (30%) and 4446 NHATS participants (41.2%) were in the cognitive impairment range. Accounting for demographic covariates and normative change in purpose, multilevel modeling indicated that standardized purpose in life declined significantly prior to onset of cognitive impairment (estimate for 10 years) in both HRS (b = −0.12; 95% CI, −0.17 to −0.07; *P* < .001) and NHATS (b = −0.10; 95% CI, −0.20 to −0.01; *P* = .03). Purpose declined significantly more rapidly following onset of cognitive impairment, with a standardized decline nearly 3 times larger compared with prior to impairment in HRS (b = −0.35; 95% CI, −0.41 to −0.29; *P* < .001) and 4 times larger in NHATS (b = −0.44; 95% CI, −0.53 to −0.34; *P* < .001).

**Conclusions and Relevance:**

In this cohort study, purpose in life declined with emergence of cognitive impairment. The decline before onset was too small to be useful to detect impending impairment in clinical settings. The steeper decline following impairment suggests that individuals are aware that their purpose is declining. Purpose may be an intervention target following cognitive impairment to maintain well-being and to reduce or slow emergence of behavioral symptoms associated with low purpose.

## Key Points

**Question:**

Is there a change in purpose in life before and after onset of cognitive impairment?

**Findings:**

This cohort study analyzed 50 985 assessments of purpose in life from 22 668 participants in the Health and Retirement Study (HRS) and 53 880 assessments from 10 786 participants in the National Health and Aging Trends Study (NHATS). Purpose declined significantly before and after the onset of cognitive impairment in HRS, with a greater decline after onset, a pattern that was replicated in NHATS.

**Meaning:**

The findings suggest that purpose in life has a small but accelerating decline during the early stages of cognitive impairment.

## Teaser

This cohort study uses data from the Health and Retirement Study and the National Health and Aging Trends Study to assess changes in purpose of life before and after onset of cognitive impairment.

## Introduction

Individuals with dementia often report feeling unmotivated to engage in activities that they once found meaningful.^1^ One antidote to this apathy may be to have a purpose in life.^2^ Purpose in life is the feeling that one’s life is meaningful and goal oriented and has direction.^3^ It is a core component of psychological well-being^4^ that has been associated consistently with better cognitive health in older adulthood.^5^ Individuals who report higher purpose, for example, perform better on cognitive tasks that measure episodic memory and verbal fluency,^6^ have less cognitive decline in older adulthood,^7^ are less likely to develop predementia syndromes,^8^ and ultimately, are at lower risk of developing incident dementia over time.^9^ Maintaining a purpose in life in dementia may help to reduce or forestall dementia-related apathy. Engagement in creative and social activities, for example, may support purpose and be associated with slower declines in health and maintaining social relationships.^10^ Encouragement of such engagement has been recommended across ages and stages of dementia.^11^

Changes in psychological function are common in dementia,^12^ and changes in personality and mood are 1 criterion for a diagnosis of dementia due to Alzheimer disease.^13^ Still, most work on psychological change across cognitive impairment has focused either on clinical markers of mental health, such as symptoms of depression or anxiety,^14^ or on trait aspects of psychological function, such as personality.^15^ Recent work, however, has indicated that caregivers perceive substantial declines in purpose in life (Cohen *d *>1.00) in care recipients after a dementia diagnosis compared with prior to diagnosis.^16^ To our knowledge, missing from this literature is the natural history of purpose in life along the continuum of dementia. Of particular interest is the timing of change in purpose across this continuum and whether it can be detected with self-report. For example, declines in purpose prior to cognitive impairment may be an early indicator of impending disease, and/or declines in purpose during cognitive impairment may be a consequence of the ongoing disease process. The 2 possibilities are not mutually exclusive.

The present research used data from 2 large, population-based longitudinal studies to examine changes in purpose in life before and during cognitive impairment while accounting for normative changes. Importantly, with numerous assessments of purpose and cognitive impairment from the same individuals tracked over time, it was possible to differentiate change before the onset of cognitive impairment from change that occurred during cognitive impairment. We included 2 longitudinal samples to ensure replicability of any identified change in purpose. We expected purpose in life to decline with cognitive impairment, particularly after impairment onset.

## Methods

### Participants and Procedure

This cohort study used data from participants in the Health and Retirement Study (HRS) from March 2006 to May 2021^17^ and the National Health and Aging Trends Study (NHATS) from May 2011 to November 2021.^18^ A random half of the HRS sample first reported their purpose in life at the 2006 assessment; the other half of the HRS sample reported their purpose in life at the 2008 assessment. Participants in HRS subsequently reported their purpose at 4-year intervals through 2020 (the most recent assessment with available data). Participants in NHATS reported their purpose at each annual assessment from the first wave in 2011 to the 2021 assessment (the most recent assessment with available data). In both studies, participants were administered a cognitive battery used to assess cognitive status at each wave. All participants with data on purpose and cognitive function at any wave during the study were included in the analysis. Scripts for the analysis are given in the eAppendix in Supplement 1. The HRS and NHATS obtained approval from their institutional review boards, and informed consent was obtained from participants prior to testing at each assessment.^17,18^ The institutional review board at the Florida State University deemed this research exempt because it was secondary research of data. The HRS and NHATS secured informed consent from participants to release the collected data to the public in deidentified form. The reporting of this research followed the Strengthening the Reporting of Observational Studies in Epidemiology (STROBE) reporting guideline.

### Measures

#### Purpose in Life

In HRS, purpose in life was assessed with the Purpose in Life subscale from the Ryff Measures of Psychological Well-Being.^3^ Participants rated 7 items (eg, “I have a sense of direction and purpose in my life”) from 1 (strongly disagree) to 6 (strongly agree). Items were reverse scored, when necessary, in the direction of greater purpose, and the mean was taken across items. In NHATS, purpose in life was measured with the item, “My life has meaning and purpose,” rated from 1 (agree a lot) to 3 (agree not at all). The item was reverse scored in the direction of greater purpose.

#### Cognitive Impairment

In HRS, cognitive function was measured with the modified Telephone Interview for Cognitive Status,^19^ which is the sum of performance on 3 cognitive tasks: word recall, serial 7s, and backward counting (possible range, 0-27). Based on a validated threshold,^19^ cognitive impairment was defined as a score less than or equal to 11. The NHATS has a standardized algorithm^20^ to classify cognitive impairment from reported diagnosis, objective cognitive function (episodic memory, orientation, and executive function), and the AD8 Dementia Screening Interview.^18^ Probable (scores ≤1.5 SDs below the mean in at least 2 cognitive domains, reported physician diagnosis of dementia, or a score ≥2 on the AD8 Dementia Screening Interview) and possible (1 cognitive domain score ≤1.5 SDs below the mean) dementia were combined into an any impairment category.^21,22^

#### Covariates

Covariates were age at baseline centered at the mean age in each study and divided by 10 to scale coefficients per decade, age squared, sex (female [1], male [0]), race (2 dummy-coded variables that compared Black [1] and otherwise identified [1; races other than Black or White, multiracial, and unknown] with White [0]), ethnicity (Hispanic or Latinx [1], not Hispanic or Latinx [0]), and education. Race and ethnicity were self-reported. In HRS, “other” is not broken down further; in NHATS, “other” is specified as American Indian, Asian, Native Hawaiian, Pacific Islander, and other. Education was reported in years in HRS and on a scale from 1 (no schooling) to 9 (graduate degree) in NHATS. These covariates were included because previous research has shown associations between these factors and both purpose in life and cognitive impairment.

### Statistical Analysis

Multilevel modeling^23^ was used to analyze the data because of the hierarchical structure of measurement occasions (level 1) nested within persons (level 2). We followed the same approach to modeling purpose over time as a previous study.^24^ Specifically, the model included intercept and slope sample means as fixed effects and individual deviations from the means as random effects (ie, variation between persons on intercepts and slopes were included in the estimated models). The covariates were included as time-invariant variables. To capture normative change in purpose in life over time,^25^ we coded time in years starting from the first assessment of purpose (eg, 0 for the first assessment, 0.4 for a second assessment 4 years later in HRS, and 0.1 for a second assessment 1 year later in NHATS); 1 unit corresponded to 1 decade for all time-related variables. We included time squared to account for a quadratic slope and a time-by-age interaction to account for differences in the slope as people aged.

Our variables of interest were 2 terms that tested change in purpose before and during cognitive impairment. The Figure shows a schematic for how time and before and during cognitive impairment were coded in NHATS for a hypothetical participant. The before cognitive impairment variable tested for change that occurred prior to the onset of cognitive impairment. It was coded with negative values for waves leading to cognitive impairment (eg, −0.4 for a purpose assessment 4 years before the wave at which a participant scored as having cognitive impairment) and 0 at waves in which participants had cognitive impairment. It is important to note that this coding did not model change at the wave in which the participant developed cognitive impairment so that the change before impairment represents change before the emergence of cognitive symptoms severe enough to identify impairment. The during cognitive impairment variable tested for change that occurred after the onset of cognitive impairment. It was coded from the first wave at which participants had cognitive impairment (eg, 0.4 for a purpose assessment completed 4 years after the wave at which participants scored as having cognitive impairment) and coded 0 for the waves before cognitive impairment. For both the before and during cognitive impairment variables, participants without cognitive impairment were the reference group and coded 0 at all waves. Supplemental analysis added a quadratic term for both variables to test for nonlinear changes in purpose both before and during impairment. Purpose in life was standardized within each sample to facilitate interpretation and comparison across the 2 studies. Two-sided *P* < .05 was considered significant. Analyses were performed using SPSS, version 28.0 (IBM Corp).

**Figure.**
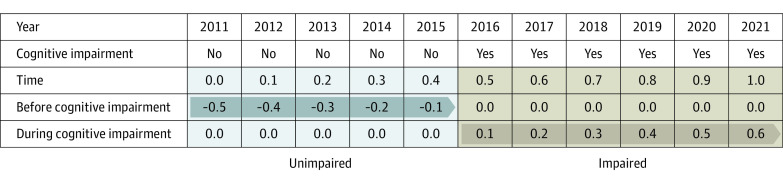
Schematic of Coding of Time and Changes Before and During Cognitive Impairment for a Participant in the National Health and Aging Trends Study Time is coded in years. Before cognitive impairment was coded with negative values leading to cognitive impairment and 0 at waves in which participants were impaired. During cognitive impairment was coded from the first wave when participants had cognitive impairment and 0 for preimpairment waves. Participants without cognitive impairment were coded as 0 at all waves and were the reference group.

## Results

In the HRS sample, 58.3% of participants were female and 41.7% were male; mean (SD) age at baseline was 64.76 (10.41) years (Table 1). A total of 17.8% of participants were Black, 74.3% were White, and 7.9% were otherwise identified race; 12.1% were Hispanic. In the NHATS sample, 57.4% of participants were female and 42.6% were male; mean (SD) age at baseline was 76.82 (7.71) years. A total of 21.3% were Black, 68.4% were White, and 10.3% were otherwise identified race; 6.1% were Hispanic. In HRS, 22 668 participants provided 50 985 assessments of purpose in life. A total of 6794 participants (30%) scored in the cognitive impairment range over the course of the study. These participants provided 3194 assessments of purpose before cognitive impairment and 8477 assessments of purpose during cognitive impairment. The unadjusted baseline means indicated that the group with cognitive impairment was older, more likely to be Black or Hispanic or Latinx, had a lower educational level, and scored lower in purpose in life compared with participants without cognitive impairment. In NHATS, 10 786 participants provided 53 880 assessments of purpose in life. A total of 4446 participants (41.2%) scored in the cognitive impairment range over the course of the study. These participants provided 4757 assessments of purpose before cognitive impairment and 8253 assessments of purpose during cognitive impairment. The unadjusted baseline means indicated that the group with cognitive impairment was older; more likely to be Black, Hispanic or Latinx, or otherwise identified race; had a lower educational level; and scored lower in purpose in life compared with participants without cognitive impairment.

**Table 1.** Baseline Descriptive Statistics for the Total Sample and by Cognitive Impairment Status Across Follow-Up

Characteristic	HRS			NHATS		
	Full sample	Unimpaired	Impaired	Full sample	Unimpaired	Impaired
Participants, No. (%)	22 668 (100)	15 874 (70.0)	6794 (30.0)	10 786 (100)	6340 (58.8)	4446 (41.2)
Assessments, No. (%)	50 985 (100)	41 439 (81.3)	9546 (18.7)	53 880 (100)	43 858 (81.4)	10 022 (18.6)
Age, mean (SD), y	64.76 (10.41)	62.87 (9.54)	69.15 (11.01)	76.82 (7.71)	74.74 (6.94)	79.78 (7.79)
Sex, No. (%)						
Female	13 226 (58.3)	9332 (58.8)	3894 (57.3)	6192 (57.4)	3677 (58.0)	2515 (56.6)
Male	9442 (41.7)	6542 (41.2)	2900 (42.7)	4594 (42.6)	2663 (42.0)	1931 (43.4)
Race, No. (%)						
Black	4033 (17.8)	2246 (14.1)	1787 (26.3)	2301 (21.3)	1145 (18.1)	1156 (26.0)
White	16 854 (74.3)	12 458 (78.5)	4396 (64.7)	7374 (68.4)	4682 (73.8)	2692 (60.5)
Otherwise identified^a^	1781 (7.9)	1170 (7.4)	611 (9.0)	1111 (10.3)	513 (8.1)	598 (13.5)
Ethnicity, No. (%)						
Hispanic or Latinx	2753 (12.1)	1635 (10.3)	1118 (16.5)	651 (6.1)	268 (4.2)	383 (8.6)
Not Hispanic or Latinx	19 915 (87.9)	14 221 (89.6)	5676 (83.5)	10 135 (94.0)	6072 (95.8)	4063 (91.4)
Educational level, mean (SD)^b^	12.74 (3.14)	13.42 (2.72)	11.16 (3.47)	5.14 (2.20)	5.58 (2.13)	4.52 (2.16)
Purpose in life, mean (SD), points^c^	4.60 (0.95)	4.68 (0.93)	4.40 (0.97)	2.81 (0.45)	2.85 (0.39)	2.74 (0.51)
Assessments, mean (SD), No.	2.25 (1.11)	2.25 (1.30)	2.24 (1.06)	5.00 (3.45)	4.91 (3.54)	5.12 (3.30)
Time, mean (SD), y	5.23 (4.58)	5.24 (4.65)	5.22 (4.41)	4.07 (0.35)	3.95 (0.36)	4.24 (0.33)

Abbreviations: HRS, Health and Retirement Study; NHATS, National Health and Aging Trends Study.

^a^ Participants who self-identified as a race other than Black or White, participants with multiracial backgrounds, and unreported race. In HRS, “other” is not broken down further; in NHATS, “other” is specified as American Indian, Asian, Native Hawaiian, Pacific Islander, and other.

^b^ Units for educational level are years in HRS and a scale that ranged from 1 (no schooling) to 9 (graduate degree) in NHATS.

^c^ Units of purpose are points on a 1- to 6-point scale for HRS and a 1- to 3-point scale for NHATS, with higher scores indicating higher purpose.

The results of the multilevel modeling analysis for both studies are given in Table 2. In HRS, after accounting for normative change in purpose over time, there was evidence for a modest change in purpose both before and during cognitive impairment. Purpose in life declined in the years preceding the development of cognitive impairment and had a steeper decline in the years during cognitive impairment. This pattern of results was replicated in NHATS. There was a modest decline in purpose prior to the development of cognitive impairment and a steeper decline during cognitive impairment. In both studies, the purpose measure was standardized and time was coded in decades. The coefficient for purpose before and during cognitive impairment can thus be interpreted as standardized change over a decade. As such, the estimated decline in purpose prior to the development of cognitive impairment over a decade was −0.12 (95% CI, −0.17 to −0.07; *P* < .001) in HRS and −0.10 (95% CI, −0.20 to −0.01; *P* = .03) in NHATS. The estimated decline in purpose during cognitive impairment over a decade was nearly 3 times greater in HRS (b = −0.35; 95% CI, −0.41 to −0.29; *P* < .001) and 4 times greater in NHATS (b = −0.44; 95% CI, −0.53 to −0.34; *P* < .001) compared with the estimated decline before the development of impairment; the estimates for change during cognitive impairment were significantly larger than those before impairment in both samples (HRS: *Z* = 26.07; NHATS: *Z* = 27.31; *P* < .001 for both). Supplemental analysis indicated that the quadratic term during impairment but not before impairment was significant, which indicated significant nonlinear change in purpose during cognitive impairment (eTable in Supplement 1). Specifically, there was accelerated change in purpose in the early years after the onset of cognitive impairment that slowed over time.

**Table 2.** Change in Purpose in Life Before and During Cognitive Impairment

Variable	HRS (N = 22 668)		NHATS (N = 10 786)	
	b (95% CI)	*P* value	b (95% CI)	*P* value
Intercept	−0.82 (−0.89 to −0.76)	<.001	−0.27 (−0.32 to −0.22)	<.001
Age^a^	−0.07 (−0.08 to −0.06)	<.001	−0.10 (−0.12 to −0.08)	<.001
Age squared^a^	−0.07 (−0.08 to −0.06)	<.001	−0.03 (−0.05 to −0.01)	.008
Female^b^	0.02 (0.00 to 0.04)	.06	0.04 (0.01 to 0.07)	.005
Race^c^				
Black	0.24 (0.21 to 0.27)	<.001	0.13 (0.09 to 0.16)	<.001
Otherwise identified	−0.02 (−0.07 to 0.02)	.37	−0.02 (−0.09 to 0.06)	.64
Hispanic or Latinx ethnicity^d^	0.08 (0.04 to 0.12)	<.001	0.02 (−0.07 to 0.12)	.64
Educational level^e^	0.06 (0.06 to 0.07)	<.001	0.04 (0.04 to 0.05)	<.001
Time^a^	−0.04 (−0.08 to 0.01)	.14	−0.08 (−.16 to 0.00)	.07
Time squared^a^	−0.01 (−0.06 to 0.03)	.53	0.02 (−0.07 to 0.12)	.67
Age × Time^a^	0.00 (−0.03 to 0.02)	.80	−0.17 (−0.23 to −0.10)	<.001
Before cognitive impairment^a^	−0.12 (−0.17 to −0.07)	<.001	−0.10 (−0.20 to −0.01)	.03
During cognitive impairment^a^	−0.35 (−0.41 to −0.29)	<.001	−0.44 (−0.53 to −0.34)	<.001

Abbreviations: HRS, Health and Retirement Study; NHATS, National Health and Aging Trends Study.

^a^ Coefficients refer to change over 10 years.

^b^ Compared with male.

^c^ Compared with White.

^d^ Compared with not Hispanic or Latinx.

^e^ Educational level was standardized, and thus, the coefficient refers to 1-SD difference in education.

## Discussion

Across 2 large samples with longitudinal assessments both before and during cognitive impairment, there was replicated evidence of modest declines in purpose in life prior to the development of cognitive impairment and significantly larger declines during cognitive impairment; purpose in life decreased about one-tenth of an SD (b = 0.12 in HRA; b = 0.10 in NHATS) in the decade before the onset of cognitive impairment and more than three-tenths an SD (b = 0.35 in HRS; b = 0.44 in NHATS) in the decade that followed onset of impairment. It is notable that the pattern of change in purpose both before and during cognitive impairment was similar across the 2 studies despite the differences between the 2 samples (eg, purpose assessed with different measures, cognitive impairment based on different classification algorithms, and difference in age). The present research describes the natural history of a critical aspect of well-being across the development and course of cognitive impairment.

Previous research on purpose in life and cognition has focused primarily on the association between purpose and healthier cognitive outcomes across older adulthood.^5^ Less work has addressed how purpose may change in association with declines in cognition. There is, however, some parallel literature on changes in apathy during the prodromal and clinical phases of Alzheimer disease. A latent class analysis of the trajectory of informant-rated apathy over time found that the largest class was no change but also identified a class of participants that had increased apathy over time.^26^ Informant-rated apathy was likewise found to increase over time among individuals with either mild cognitive impairment^27^ or dementia.^28^ The decline in purpose found in the current study is consistent with this literature. It may be that as the drive to be goal focused and future oriented declines, lack of motivation and feelings of emptiness may increase.

The largest decline in purpose in life occurred following onset of cognitive impairment. This greater change may be due in part to the neurodegeneration that causes cognitive impairment.^29^ That is, decline in purpose may be an additional consequence of neurodegeneration in addition to deficits in cognition. There are also changes in activity participation^30^ and social integration^31^ that occur as cognitive deficits limit the routine activities individuals can engage in. Furthermore, studies of retrospective change in purpose measured with informant ratings indicated that purpose declined sharply with increasing severity of dementia.^16^ There may thus be accelerated change as neurodegeneration becomes more severe.

Interestingly, changes in both purpose^16^ and apathy^26,27,28^ found in previous studies that used informant ratings were larger in magnitude than the changes observed in self-reported purpose in the current study. There are at least 3 non–mutually exclusive reasons for this difference. First, cognitive impairment in the current study was likely to be mild because participants were still active in HRS and NHATS and able to report their own feelings of purpose. Purpose may have greater declines later in the disease process. Second, individuals with cognitive impairment may have anosognosia and may not be able to update their perceptions of themselves and, thus, may rely on self-perceptions prior to impairment, whereas informants may have a better sense of changes that have occurred with impairment. Third, informants could generalize instances of low purpose or apathy to be characteristic of the person with dementia and/or exaggerate changes compared with prior to impairment.

There is great interest in identifying psychological changes before the onset of cognitive deficits to identify individuals who may be about to develop cognitive impairment.^32^ The present research suggests that change in self-reported purpose prior to impairment is likely too modest for practical use to detect an imminent impairment; the small change is unlikely to be perceptible on standard measures in clinical settings. The decline prior to impairment also has relevance for interpreting the association between purpose in life and risk of cognitive impairment. That is, the association often found between purpose and lower risk of incident dementia may reflect early manifestation of the disease process rather than being a protective factor. There are at least 2 reasons to doubt this reverse causality. First, the association between purpose and dementia risk may be similar regardless of length of follow-up.^9^ The risk should be higher with shorter follow-up if it is due solely to the disease process. Second, in 1 study,^33^ the effect size for change in purpose prior to dementia was smaller than the mean difference in purpose at baseline between those who did and did not develop dementia (Cohen *d* = 0.35). The modest change prior to dementia (Cohen *d* = 0.1) would not account for this difference.

The present research contributes to general knowledge on purpose in life, particularly its natural history in the context of cognitive impairment. Given its importance for both well-being in general^4^ and better outcomes even after the onset of dementia specifically,^34^ purpose in life should be preserved and cultivated following the onset of impairment. This issue may become especially important when effective treatments for dementia are discovered and patients may need help in recovering their sense of purpose, especially as they recover cognitive function.^35^ Purpose can be increased through engagement in goal-directed activities among individuals with dementia.^36^ Focused engagement in creative and social activities may also help to support purpose.^10^ Such approaches may have the dual utility of supporting well-being and reducing apathy and other behavioral symptoms that may contribute to faster progression of dementia. It will be critical for future research to test how purpose can be supported with onset of cognitive impairment and how to best identify and support purpose during cognitive recovery.^35^

### Strengths and Limitations

This research had several strengths, including the use of 2 independent samples, the large size of each sample, and the repeated longitudinal assessments of purpose in life both before and during cognitive impairment. In particular, the replication across 2 samples reduced the likelihood that the declines observed were due to chance.

There are also some limitations, including the use of performance-based measures to identify cognitive impairment, the focus on early stages of cognitive impairment, and that both samples were from the US, which may limit generalizability. Another limitation is the limited number of assessments, especially in HRS, and the quadratic terms should be interpreted considering this limitation. Future research could use a clinical diagnosis of mild cognitive impairment or dementia, obtain observer ratings to track changes later in the disease continuum, and examine samples from more diverse geographic locations.

## Conclusions

In this cohort study, we found replicable declines in purpose in life, one of the major dimensions of well-being, across the preclinical and clinical stages of cognitive impairment. The decline was greater during cognitive impairment. In clinical settings, change in purpose in life may be too modest to use to identify incident cases of impairment before the onset of cognitive deficits. Interventions to maintain purpose may help support psychological and cognitive health both before and during cognitive impairment.

## Notes

Supplement 1. **eTable.** Quadratic change in purpose in life before and during cognitive impairment
eAppendix.

Supplement 2.
Data Sharing Statement
