# Supplementary material for: Change in Purpose in Life Before and After Onset of Cognitive Impairment
Source: JAMA Netw Open. 2023 Sep 13;6(9):e2333489. doi: 10.1001/jamanetworkopen.2023.33489 (PMC10500383; doi:10.1001/jamanetworkopen.2023.33489)
Supplement: Supplement 1. — eTable. Quadratic change in purpose in life before and during cognitive impairment eAppendix. [file jamanetwopen-e2333489-s001.pdf]

Supplemental Online Content

Sutin AR, Luchetti M, Stephan Y, Terracciano A. Change in purpose in life before and after onset of cognitive impairment. *JAMA Netw Open.* 2023;6(9):e2333489. doi:10.1001/jamanetworkopen.2023.33489

eTable. Quadratic change in purpose in life before and during cognitive impairment

eAppendix.

This supplemental material has been provided by the authors to give readers additional information about their work.

© 2023 Sutin AR et al. *JAMA Network Open.*

eTable. Quadratic change in purpose in life before and during cognitive impairment

Predictor	HRS			NHATS		
	b	95% CI	p	b	95% CI	p
Intercept	-0.78	-0.85, -0.76	<.001	-0.25	-0.30, -0.20	<.001
Age	-0.06	-0.07, -0.06	<.001	-0.09	-0.12, -0.07	<.001
Age^2	-0.07	-0.08, -0.06	<.001	-0.03	-0.05, -0.01	0.010
Sex (female)	0.02	0.00, 0.04	0.086	0.04	0.01, 0.07	0.005
Race (Black)	0.25	0.22, 0.28	<.001	0.13	0.09, 0.17	<.001
Race (Otherwise identified)	-0.01	-0.06, 0.03	0.536	-0.01	-0.09, 0.06	0.715
Ethnicity (Hispanic/Latinx)	0.08	0.04, 0.12	<.001	0.02	-0.07, 0.12	0.620
Education	0.06	0.06, 0.06	<.001	0.04	0.04, 0.05	<.001
Time	0.00	-0.04, 0.05	0.957	-0.05	-0.14, 0.02	0.177
$Time^2$	-0.06	-0.10, -0.01	0.015	-0.02	-0.11, 0.08	0.732
Age*Time	0.00	-0.03, 0.02	0.852	-0.17	-0.24, -0.10	<.001
Before-Cognitive impairment	-0.14	-0.27, -0.02	0.027	-0.01	-0.23, 0.21	0.902
Before-Cognitive $impairment^2$	-0.10	-0.22, 0.03	0.133	0.06	-0.24, 0.37	0.684
During-Cognitive impairment	-1.05	-1.20, -0.90	<.001	-0.79	-0.98, -0.60	<.001
During-Cognitive $impairment^2$	0.76	0.62, 0.91	<.001	0.53	0.30, 0.77	<.001

Note. N=22668 for HRS and N=10786 for NHATS. HRS=Health and Retirement Study. NHATS=National Health and Aging Trends Study. CI=confidence interval. Age and time (including before and during cognitive impairment) are in decades such that the coefficients refer to change over 10 years. Sex compares females to males. The race variables compare Black and Otherwise identified participants to White participants. Education is standardized and thus the coefficient refers to one standard deviation difference in education.

© 2023 Sutin AR et al. *JAMA Network Open.*

eAppendix.
Sample SPSS syntax for analysis of HRS:

```
MIXED Zpurposelife WITH AGE_68 AGE_SQUARED RAGENDER educ_nomiss race_AA_nomiss
race_other_nomiss Latinx_nomiss
 time.decade time.sq.decade  to_impaired.yr     post_impaired.yr
   /CRITERIA=DFMETHOD(SATTERTHWAITE) CIN(95) MXITER(100) MXSTEP(10) SCORING(1)
    SINGULAR(0.000000000001) HCONVERGE(0, ABSOLUTE) LCONVERGE(0, ABSOLUTE)
PCONVERGE(0.000001, ABSOLUTE)
  /FIXED=AGE_68 AGE_SQUARED RAGENDER educ_nomiss race_AA_nomiss race_other_nomiss
Latinx_nomiss
  time.decade time.sq.decade  time.decade*AGE_68      to_impaired.yr     post_impaired.yr  |
SSTYPE(3)
  /METHOD=ML    /PRINT= DESCRIPTIVES SOLUTION  /RANDOM=INTERCEPT time.decade |
SUBJECT(HHIDPN) COVTYPE(VC).
```

Sample SPSS syntax for analysis of NHATS:

```
MIXED Zpurpose WITH AGE_79 AGE_SQUARED sex  educ race_d1 race_d2 hisp
 time time.sq   To_impairment     During_impairment
   /CRITERIA=DFMETHOD(SATTERTHWAITE) CIN(95) MXITER(100) MXSTEP(10) SCORING(1)
    SINGULAR(0.000000000001) HCONVERGE(0, ABSOLUTE) LCONVERGE(0, ABSOLUTE)
PCONVERGE(0.000001, ABSOLUTE)
  /FIXED=AGE_79 AGE_SQUARED sex educ race_d1 race_d2 hisp
 time time.sq AGE_79*time  To_impairment     During_impairment  | SSTYPE(3)
  /METHOD=ML    /PRINT= DESCRIPTIVES SOLUTION  /RANDOM=INTERCEPT time |
SUBJECT(SPID) COVTYPE(VC).
```

© 2023 Sutin AR et al. *JAMA Network Open.*
