# Supplementary material for: Change in Purpose in Life Before and After Onset of Cognitive Impairment
Source: JAMA Netw Open. 2023 Sep 13;6(9):e2333489. doi: 10.1001/jamanetworkopen.2023.33489 (PMC10500383; doi:10.1001/jamanetworkopen.2023.33489)
Supplement: Supplement 2. — Data Sharing Statement [file jamanetwopen-e2333489-s002.pdf]

Data Sharing Statement

Sutin. Change in Purpose in Life Before and After Onset of Cognitive Impairment. *JAMA Netw Open*. Published September 13, 2023. doi:10.1001/jamanetworkopen.2023.33489

Data
Data available: Yes
Data types: Deidentified participant data, Data dictionary
How to access data: Deidentified data are available to the public from the parent studies at https://hrs.isr.umich.edu/about (HRS) and https://www.nhats.org/researcher (NHATS)
When available: With publication

Supporting Documents
Document types: Statistical/analytic code
How to access documents: In supplemental material
When available: With publication

Additional Information
Who can access the data: Deidentified data are available to the public from the parent studies at https://hrs.isr.umich.edu/about (HRS) and https://www.nhats.org/researcher (NHATS)
Types of analyses: any purpose
Mechanisms of data availability: Deidentified data are available to the public from the parent studies at https://hrs.isr.umich.edu/about (HRS) and https://www.nhats.org/researcher (NHATS)
